# Efficacy in Japanese adults with growth hormone deficiency receiving weekly somapacitan or daily growth hormone: results from phase 3 REAL 1 trial

**DOI:** 10.3389/fendo.2025.1534891

**Published:** 2025-02-07

**Authors:** Fumio Otsuka, Michael Højby Rasmussen, Takaaki Endo, Claus Sværke, Shigeyuki Tahara, Gudmundur Johannsson

**Affiliations:** ^1^ Department of General Medicine, Okayama University Graduate School of Medicine, Dentistry and Pharmaceutical Sciences, Okayama, Japan; ^2^ Clinical Drug Development, Novo Nordisk A/S, Søborg, Denmark; ^3^ Medical Affairs Department, Novo Nordisk Pharma Ltd., Tokyo, Japan; ^4^ Department of Neurological Surgery, Nippon Medical School, Musashikosugi Hospital, Kawasaki City, Kanagawa, Japan; ^5^ Department of Internal Medicine and Clinical Nutrition, Institute of Medicine at Sahlgrenska Academy, University of Gothenburg and Department of Endocrinology, Diabetology, and Metabolism, Sahlgrenska University Hospital, Göteborg, Sweden

**Keywords:** growth hormone, adult growth hormone deficiency, long-acting growth hormone, somapacitan, Japan

## Abstract

**Introduction:**

Somapacitan is a long-acting growth hormone (GH) derivative approved for once-weekly treatment of adult GH deficiency (AGHD). This study aimed to evaluate the efficacy and safety of somapacitan and daily GH in Japanese individuals with AGHD.

**Methods:**

This subgroup study included the 34-week main period of the multinational, randomized, parallel-group, phase 3 trial, REAL 1 (NCT02229851). Participants received once-weekly somapacitan or daily GH (Norditropin^®^), both administered subcutaneously. Investigators and trial sites were blinded. A total of 36 Japanese GH-naïve individuals with AGHD were allocated to somapacitan or daily GH (excluding the placebo group of the global trial). Endpoints included change in truncal fat percentage to week 34 measured using dual-energy x-ray absorptiometry, as well as other body composition measures. Insulin-like growth factor I standard deviation score values were used for dose titration.

**Results:**

In total, 36 Japanese individuals were included (18 and 18 in the somapacitan and daily GH group, respectively). 35 completed the main period (34 weeks). Mean (SD) change from baseline to week 34 in truncal fat percentage was -2.23 (2.50) %-points in the somapacitan group and -2.12 (4.21) %-points in the daily GH group. Other body composition measures also improved in both groups, including reduced visceral fat and increased lean body mass. Somapacitan was well tolerated, with a safety profile similar to daily GH.

**Conclusions:**

In Japanese treatment-naïve individuals with AGHD, once-weekly somapacitan demonstrated similar efficacy and safety to daily GH after 34 weeks of treatment. Somapacitan provides an effective alternative to daily GH in AGHD.

**Clinical trial registration:**

http://www.clinicaltrials.gov, identifier NCT02229851.

## 1 Introduction

Adult growth hormone deficiency (AGHD) is a complex condition that affects the body’s metabolism and results in unfavorable body composition, including increased abdominal fat and decreased lean body mass, as well as decreased bone mineral density (1, 2). AGHD due to hypopituitarism with this metabolic syndrome-like phenotype can lead to impaired physical activity and poor quality of life (1–3), and has been associated with increased cardiovascular and cerebrovascular morbidity, leading to premature mortality (4). Growth hormone (GH) replacement therapy is used to counteract the detrimental effects of AGHD. However, the daily injection regimen can be burdensome for patients, leading to treatment fatigue, and consequently, reduced adherence (5). The recent development of long-acting GH formulations for once-weekly injection regimen presents an alternative that potentially improve patient adherence and persistence, thus optimizing clinical efficacy, and lowering the barrier to initiating and maintaining replacement therapy (6).

Somapacitan (Novo Nordisk A/S, Denmark) is a long-acting reversible albumin-binding GH derivative, approved for once-weekly administration to treat growth hormone deficiency (GHD) in children and adults. Somapacitan contains a small albumin-binding moiety (1,200 Da), which facilitates reversible binding to circulating endogenous albumin, thereby extending the half-life and allowing once-weekly administration. Randomized controlled trials have demonstrated similar efficacy, tolerability, and safety profile of somapacitan and daily GH for AGHD as well as GHD in children (7–12), but with somapacitan reported to be more convenient with reduced treatment burden compared to daily GH (8, 9, 12).

The efficacy and safety of somapacitan in individuals with AGHD was confirmed in the large multinational, phase 3 trial, REAL 1, randomizing 301 GH treatment-naïve individuals with AGHD to once-weekly somapacitan, daily GH, or once-weekly placebo (13). After 34 weeks, once-weekly somapacitan demonstrated superiority over placebo on several body composition measures, including the primary endpoint change in truncal fat percentage, and it was concluded that somapacitan may be an effective alternative to daily GH in AGHD. Even though the trial was not powered to compare the two active treatment arms, a less pronounced effect of somapacitan compared with daily GH was noted for the reduction in truncal fat percentage. This could not be explained by different responses in insulin-like growth factor I (IGF-I) standard deviation score (SDS), as mean IGF-I SDS and IGF-I SDS distribution were similar between treatment groups. Since the REAL 1 trial was conducted in 17 countries, with stratified randomization according to region (Japan/all other countries), investigating the Japanese subgroup could provide important insights whether this finding seems to persist in a more homogeneous subpopulation. One phase 3 trial, that was conducted in previously GH treated Japanese individuals with AGHD, found that somapacitan demonstrated similar efficacy to daily GH on adipose tissue endpoints, measured using CT, and a similar sustained IGF-I profile (9). The Japanese subgroup in the pivotal REAL 1 trial could potentially extend such knowledge to GH treatment-naïve Japanese individuals with AGHD.

Hence, we present novel results of a subgroup study for the Japanese subpopulation in the somapacitan and daily GH groups from the REAL 1 trial, to evaluate the efficacy and safety of somapacitan and daily GH in GH treatment-naïve Japanese individuals with AGHD.

## 2 Materials and methods

### 2.1 Study design

The REAL 1 was a multicenter, multinational, randomized, parallel-group, placebo- and active-controlled phase 3 trial conducted at 92 sites in 17 countries in Africa, Asia, Europe, North America, and Australia (ClinicalTrials.gov: NCT02229851). Participants were randomized in a 2:1:2 ratio to receive either once-weekly somapacitan, once-weekly placebo, or daily GH for 34 weeks, followed by an extension period. The randomization was stratified by region (Japan/all other countries), sex, and diabetic status, and was carried out via a trial-specific, web-based interactive voice/web response system. The main trial period consisted of dose titration for 8 weeks followed by fixed-dose treatment for 26 weeks. The main trial period was double-blind with respect to once-weekly somapacitan and placebo, but open-label with respect to daily GH. The investigators and trial sites remained blinded throughout the trial. The protocol was approved by local and national ethics committees, as appropriate, and conducted in accordance with the International Conference on Harmonisation guidelines for Good Clinical Practice and the Declaration of Helsinki. Written informed consent was obtained from all patients prior to inclusion. Further information about the REAL 1 trial was previously published (13). In this subgroup study, we will report the results of the main trial period for the Japanese subpopulation in the somapacitan and daily GH groups. The placebo group will not be considered in this analysis, as the primary results, comparing somapacitan to placebo after 34 weeks, has already been reported separately for the global pivotal REAL 1 trial (13).

### 2.2 Participants

In Japan, screening was performed at 15 sites, with 14 sites randomizing participants. In brief, key inclusion criteria were: age 23 to 79 years, confirmed diagnosis of adult- or childhood-onset GHD, naïve to GH treatment or no exposure for at least 180 days prior to randomization, IGF-I SDS < -0.5 at screening, any replacement therapies for other hormone deficiencies adequate and stable, adequate testosterone level (males only), free T4 levels within normal range, and adequate adrenal function. Furthermore, for Japan, diabetes was among the exclusion criteria. A detailed description of inclusion and exclusion criteria was previously published (13).

### 2.3 Study drug administration and dose selection

Both somapacitan (Novo Nordisk A/S, Denmark) and daily GH (somatropin; Norditropin FlexPro^®^; Novo Nordisk A/S, Denmark) were administered by subcutaneous injections. Starting doses of somapacitan were 1.5 mg/week for participants aged 23 to 60 years, 1.0 mg/week for participants aged > 60 years, and 2.0 mg/week for female participants on oral estrogen irrespective of age (equivalent to 0.21, 0.14, and 0.29 mg/day, respectively). Starting doses for daily GH were 0.2, 0.1, and 0.3 mg/day, respectively. The individual dose titrations were done according to an algorithm to achieve a steady state IGF-I SDS target of −0.50 to +1.75. The minimum and maximum doses were set to 0.1 and 8 mg/week for somapacitan, and to 0.05 and 1.0 mg/day for daily GH. Individual dose levels were fixed after week 8 but could be reduced at the investigator’s discretion for safety concerns.

### 2.4 Efficacy assessments

Dual-energy x-ray absorptiometry (DXA)-derived body composition measures included truncal fat mass, visceral fat, total fat mass, gynoid fat mass, android fat mass, total lean body mass, truncal lean body mass, and appendicular skeletal muscle mass, and were measured at baseline and week 34. In the global REAL 1 trial, change from baseline to week 34 in truncal fat percentage (defined as 100 times truncal fat mass divided by the sum of truncal fat mass and truncal lean body mass) was the primary efficacy endpoint. Further endpoints included IGF-I SDS and insulin-like growth factor binding protein-3 (IGFBP-3) SDS, and IGF-I/IGFBP-3 molar ratio, and were measured at baseline and weeks 1, 2, 3, 4, 5, 7, 8, 9, 16, 25, and 33. For somapacitan, the samplings at baseline and weeks 2, 4, and 8 were intended to be done before dosing (i.e., around trough level), whereas the samplings at weeks 1, 3, 5, 7, 9, 16, 25, and 33 were intended to be done between planned doses (i.e., around average level), as described previously (13). IGF-I/IGFBP-3 molar ratio was calculated *post hoc* as described by Friedrich et al., 2014 (14).

### 2.5 Safety assessments

Safety was assessed by the incidence of adverse events (AEs) and serious AEs, which were summarized by treatment, Medical Dictionary for Regulatory Activities (MedDRA) system organ class, and MedDRA preferred term. Assessment of antibodies against somapacitan (somapacitan group) or GH (daily GH group) was performed by the study sponsor using a validated anti-somapacitan or anti-human GH antibody-binding assay. Glycated hemoglobin A1c (HbA_1c_) was also assessed.

### 2.6 Statistical analysis

In this subgroup study, all efficacy and safety endpoints were analyzed using descriptive statistics. The full analysis set, used to evaluate efficacy endpoints, included all randomized participants who received at least one dose of randomized treatment. In this study, the full analysis set and the safety analysis set, used to evaluate safety endpoints, were identical.

Treatment compliance was assessed using the treatment doses recorded in the participant’s diary. Adherence was calculated as the number of doses that participants reported taking divided by the number of prescribed doses and multiplied by 100.

Furthermore, we conducted supplementary analyses estimating mean changes adjusted for baseline values, estimated from an analysis of covariance model (ANCOVA) with treatment, GHD onset type, and sex as factors and baseline as a covariate.

## 3 Results

### 3.1 Study population

A total of 36 Japanese participants were randomized to once-weekly somapacitan (n=18) or daily GH (n=18). These participants were extracted from the global trial population, which was stratified by region (Japan vs. Rest-of-world). Therefore, the number of Japanese participants randomized equally to each treatment (daily GH and once-weekly somapacitan) is expected to be similar. Likewise, the treatment groups are expected to have similar baseline characteristics. At week 34, 18 (100%) had completed treatment in the somapacitan group and 17 (94%) had completed treatment in the daily GH group. Only one participant discontinued treatment due to AE (a case of diabetes in the daily GH group).

Baseline characteristics are shown in **Table 1** and were similar among the groups. Compared to the global trial (13), the Japanese participants generally had a lower mean body weight, BMI, and waist circumference, and fewer women were on oral estrogen. Oral estrogen was used in doses of 0.6 to 1.3 mg. Furthermore, a higher proportion had childhood onset GHD in the global trial.

**Table 1:** Participant characteristics at baseline.

Characteristics	Somapacitan (n = 18)	Daily GH (n = 18)	P-value
Age, years	52.6 (13.2)	51.8 (12.4)	0.86
Sex:			0.87
Female, n (%)	9 (50%)	8 (44%)	
Male, n (%)	9 (50%)	10 (56%)	
Race:			na
Asian, n (%)	18 (100%)	18 (100%)	
Body weight, kg	61.5 (11.2)	68.0 (15.9)	0.17
BMI, kg/m^2^	23.8 (3.1)	24.5 (4.3)	0.55
Waist circumference, cm	86.1 (9.1)	88.7 (10.7)	0.43
IGF-I SDS	-2.13 (1.11)	-2.13 (0.84)	1.00
IGFBP-3 SDS	-1.14 (1.42)	-1.13 (1.24)	0.97
IGF-I/IGFBP-3 molar ratio	7.10 (1.90)	7.06 (1.94)	0.96
Using oral estrogen, n (%)	3 (17%)^b^	1 (6%)^b^	0.35
GHD Onset:			0.39
Childhood - idiopathic, n (%)	1 (6%)	0	
Childhood - organic, n (%)	2 (11%)	1 (6%)	
Adulthood, n (%)	15 (83%)	17 (94%)	
Type of GHD:			0.53
GHD only, n (%)	0	1 (6%)	
Multiple pituitary hormone deficiency^a^, n (%)	18 (100%)	17 (94%)	

Values are mean (SD), unless otherwise indicated.

a Multiple pituitary hormone deficiency was defined in the same way as in the global trial, i.e., as GHD plus at least one other deficiency (terms reported: panhypopituitarism, empty sella syndrome, Sheehan syndrome, single axis deficiencies), or if concomitant medication included treatment for deficiency of one or more pituitary axes.

b Percentages presented are calculated based on the number of participants in each group. Percentages calculated based on the number of females in each group are 3/9 (33%) and 1/8 (13%), respectively. BMI, body mass index; GH, growth hormone; GHD, growth hormone deficiency; IGF-I, insulin-like growth factor I; IGFBP-3, insulin-like growth factor binding protein-3; SD, standard deviation; SDS, standard deviation score.

NA, Not applicable.

Median exposure was 238 days in both groups, and the mean (SD) treatment dose was 1.71 (1.09) mg/week in the somapacitan group and 0.24 (0.10) mg/day (equivalent to 1.68 mg/week) in the daily GH group. Median treatment adherence among the participants was 100% for somapacitan and 99.6% for daily GH. Mean (SD) treatment adherence was 99.0% (2.85) for somapacitan and 99.2% (1.24) for daily GH.

### 3.2 Body composition

**Figure 1:**
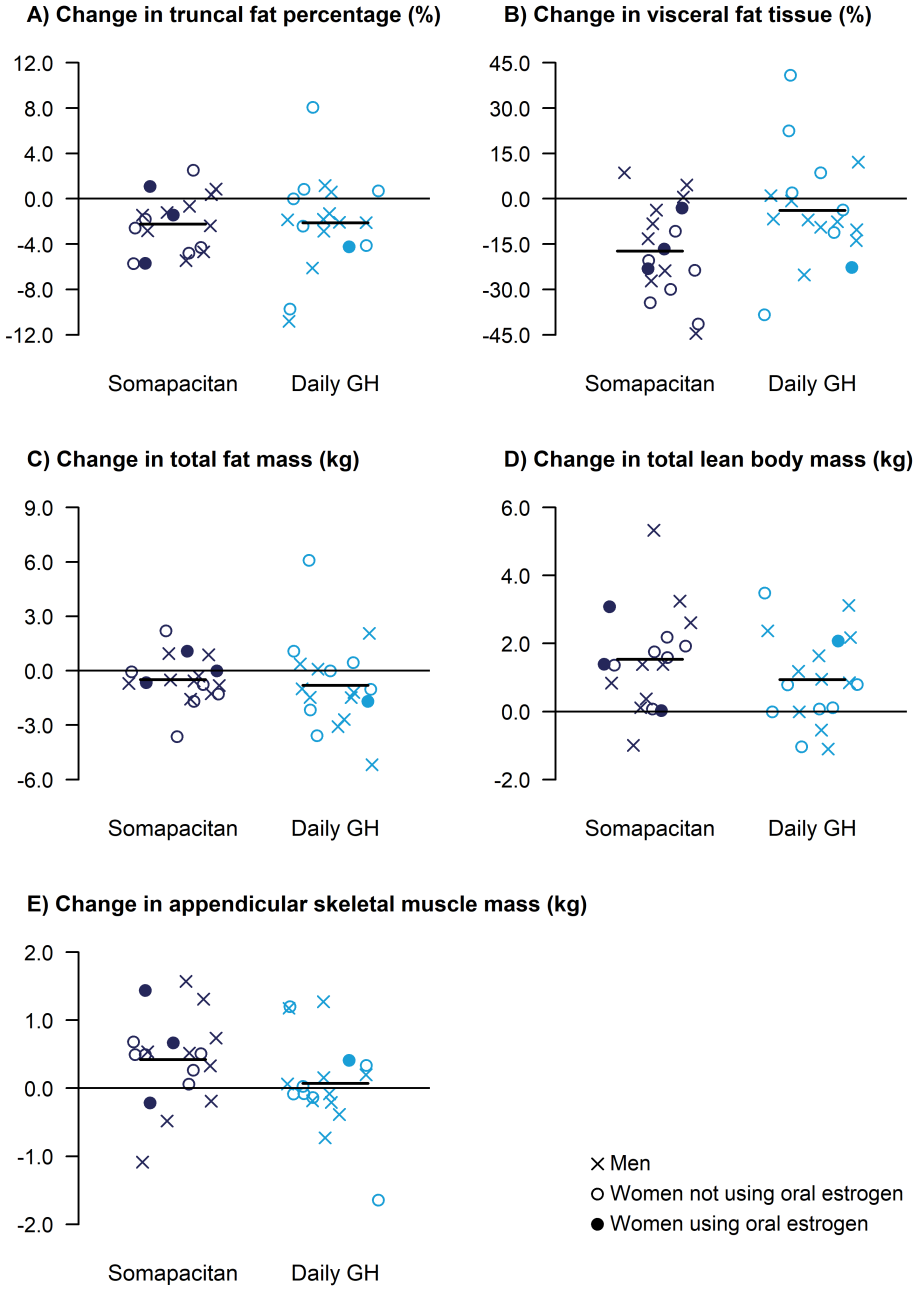
Changes from baseline in DXA-derived body composition measures at 34 weeks, including truncal fat percentage **(A)**, visceral fat tissue **(B)**, total fat mass **(C)**, total lean body mass **(D)**, and appendicular skeletal muscle mass **(E)**. For effects on fat mass, a reduction is desired. For effects on lean mass, an increase is desired. Data are presented as change values for each individual participants, with mean change shown as horizontal lines. DXA, dual-energy x-ray absorptiometry; GH, growth hormone.

The body weight mean change (SD) from baseline to week 34 was 1.2 (1.5) and -0.6 (2.7) kg in the somapacitan and daily GH group, respectively. The mean (SD) change in truncal fat percentage at week 34 was similar between the groups, with -2.23 (2.50) %-points for somapacitan and -2.12 (4.21) %-points for daily GH (**Figure 1A**; **Table 2**). Improvements in body composition measures were seen in both the somapacitan and daily GH groups. Mean reductions from baseline in the measures for fat mass were similar (**Figures 1A, C**) except for visceral fat (**Figure 1B**), where the somapacitan group appeared to show more pronounced decreases compared to the daily GH group (**Figure 1B**; **Table 2**). For the measures for lean mass, the mean increases from baseline appeared slightly more pronounced in the somapacitan group compared to the daily GH group (**Figures 1D, E**; **Table 2**). When adjusting the mean changes for baseline values, a similar pattern was seen, but the body composition measures became more similar between the groups (**Supplementary Table S1**). Furthermore, there were no apparent patterns indicating that men, women not using oral estrogen, or women on oral estrogen showed distinct effects on body composition measures (**Figure 1**).

**Table 2:** Baseline values and observed changes from baseline to week 34 in DXA-derived body composition measures.

	Somapacitan (n = 18)	Daily GH (n = 18)
Effects on total body weight		
Total body weight, kg		
Baseline	61.5 (11.2)	68.0 (15.9)
Change	1.2 (1.5)	-0.6 (2.7)
Percentage change	2.0 (2.4)	-0.5 (3.8)
Effects on fat mass		
Truncal fat percentage, %		
Baseline	33.06 (8.86)	32.42 (5.17)
Change	-2.23 (2.50)	-2.12 (4.21)
Percentage change in visceral fat^a^		
Baseline	100.00	100.00
Change	-17.27 (15.36)	-3.87 (17.87)
Total fat mass, kg		
Baseline	18.31 (4.63)	21.17 (6.43)
Change	-0.49 (1.28)	-0.81 (2.46)
Truncal fat mass, kg		
Baseline	9.89 (3.00)	10.96 (3.67)
Change	-0.53 (0.75)	-0.42 (1.60)
Gynoid fat mass, kg		
Baseline	2.85 (0.72)	3.39 (0.98)
Change	-0.02 (0.29)	-0.11 (0.48)
Android fat mass, kg		
Baseline	1.66 (0.57)	1.86 (0.72)
Change	-0.16 (0.17)	-0.11 (0.26)
Effects on lean mass		
Total lean body mass, kg		
Baseline	40.91 (10.42)	44.43 (10.68)
Change	1.53 (1.46)	0.94 (1.33)
Truncal lean body mass, kg		
Baseline	20.33 (5.57)	22.51 (5.22)
Change	1.04 (0.93)	0.84 (0.81)
Appendicular skeletal muscle mass, kg		
Baseline	17.60 (4.92)	19.10 (5.33)
Change	0.42 (0.66)	0.07 (0.69)

Values are mean (SD). Efficacy results from the global REAL1 population have previously been published (13).

a *Post hoc* defined endpoint. Changes are expressed as a percentage, therefore initial values are 100%.

DXA, dual-energy x-ray absorptiometry; GH, growth hormone.

### 3.3 IGF-I SDS and IGFBP-3 SDS

**Figure 2:**
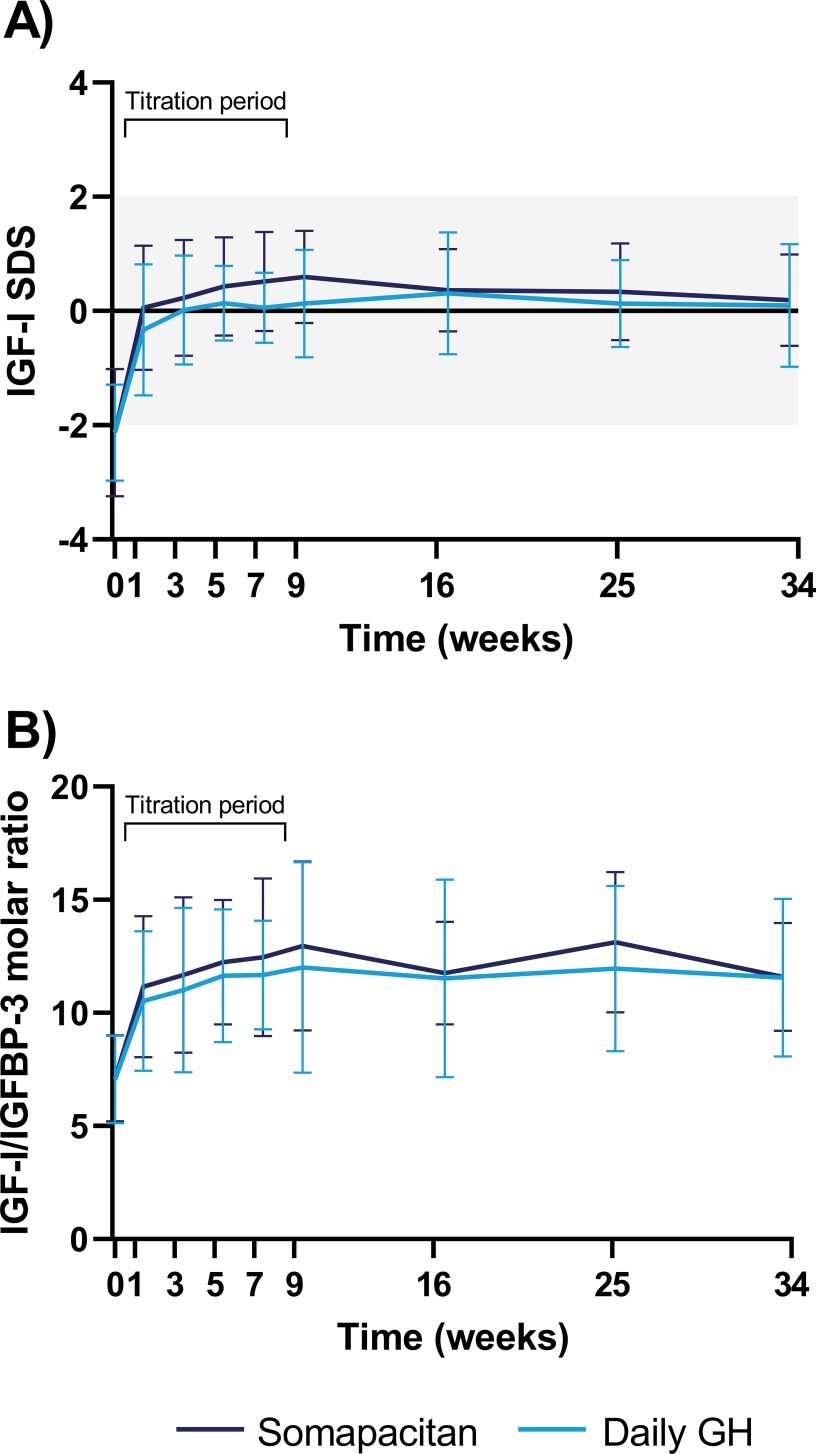
IGF-I SDS **(A)** and IGF-I/IGFBP-3 molar ratio **(B)** from baseline to week 34. Data are presented as mean with error bars representing SD. The grey area in panel **(A)** indicates the intended normal reference range (-2 to +2 SDS). For visual purposes, trough IGF-I samples are not included. The first 8 weeks were a titration period to achieve a steady state IGF-I SDS target of -0.50 to +1.75. GH, growth hormone; IGF-I, insulin-like growth factor I; IGFBP-3, insulin-like growth factor binding protein-3; SD, standard deviation; SDS, standard deviation score.

At baseline, the groups had similar mean (SD) IGF-I SDS, with values of -2.13 (1.11) and -2.13 (0.84) in the somapacitan group and daily GH group, respectively. At week 34, all participants in the somapacitan group, and all except one in the daily GH group, were within the intended normal reference range of -2 to +2, and the groups were still similar, with mean (SD) values of 0.19 (0.80) in the somapacitan group and 0.10 (1.08) in the daily GH group (**Figure 2A**). A similar pattern was seen for IGFBP-3 SDS, with mean (SD) values at week 34 of 0.27 (1.25) in the somapacitan group and 0.14 (1.25) in the daily GH group. IGF-I/IGFBP-3 molar ratio increased during the titration period and subsequently stabilized, with mean (SD) values at week 34 of 11.59 (2.38) in the somapacitan group and 11.56 (3.49) in the daily GH group (**Figure 2B**). The mean (SD) values at each visit for IGF-I SDS, IGFBP-3 SDS, and IGF-I/IGFBP-3 are provided in the **Supplementary Table S2**. Two participants in the daily GH group had transient IGF-I SDS values above +2 at one time point each during the fixed-dose treatment period (weeks 9–34).

### 3.4 Safety

A total of 38 AEs were reported by 11 (61%) participants in the somapacitan group, and 46 events were reported by 18 (100%) participants in the daily GH group (**Table 3**). The majority of the events were mild and assessed to be unlikely related to the trial product. All 38 AEs reported in the somapacitan group were reported as recovered, whereas 33 of the 46 AEs reported in the daily GH group were recovered. The most frequent AE was common cold (nasopharyngitis). Non-frequent AEs (occurring in only one participant in any treatment arm) that were rated probably or possibly related to the trial product included dizziness, somnolence, diarrhea and upper abdominal pain in the somapacitan group, and limb discomfort, pain in extremity, face edema, fat tissue decreased, diabetes, and subcutaneous hemorrhage in the daily GH group, as well as a case of back pain in both groups.

**Table 3:** Adverse events.

	Somapacitan (n = 18)			Daily GH (n = 18)		
	N (%)	E	R	N (%)	E	R
**All adverse events**	11 (61)	38	313.4	18 (100)	46	379.4
**Serious adverse events**	0			1 (6)	1	8.2
Severity						
Mild	11 (61)	36	297.0	16 (89)	41	338.2
Moderate	2 (11)	2	16.5	4 (22)	4	33.0
Severe	0			1 (6)	1	8.2
Relation to trial product^a^						
Probable	2 (11)	2	16.5	6 (33)	7	57.7
Possible	2 (11)	7	57.7	3 (17)	4	33.0
Unlikely	10 (56)	29	239.2	16 (89)	35	288.7
Most frequent adverse events^b^						
*Infections and infestations*						
Nasopharyngitis	6 (33)	9	74.2	9 (50)	10	82.5
Tonsillitis	2 (11)	2	16.5	0		
*Musculoskeletal and connective tissue disorders*						
Arthralgia	2 (11)	2	16.5	2 (11)	2	16.5
Pain in extremity	0			3 (17)	3	24.7
*General disorders and administration site conditions*						
Oedema peripheral	2 (11)	2	16.5	2 (11)	2	16.5
*Gastrointestinal disorders*						
Dental caries	0			2 (11)	2	16.5
Vomiting	2 (11)	2	16.5	0		
*Nervous system disorders*						
Headache	1 (6)	1	8.2	2 (11)	2	16.5

a Relation to trial product (i.e., causality) is based on judgement of investigators.

b Most frequent adverse events occurring in ≥10% of participants (i.e., at least two) in any treatment arm, organized according to MedDRA system organ class preferred term.

%, percentage of participants; E, number of events; GH, growth hormone; MedDRA, Medical Dictionary for Regulatory Activities; N, number of participants having the given event at least once; R, event rate per 100 patient-years at risk.

Serious AEs were reported for only one participant in the daily GH group. The serious AE was a case of multiple myeloma, assessed to be severe but unlikely to be related to the trial product. It occurred on study day 146, but was diagnosed after week 34, and therefore did not lead to treatment discontinuation. There were no treatment discontinuations due to AEs in the somapacitan group. In the daily GH group, there was one treatment discontinuation due to AEs on study day 177. It was a case of diabetes, assessed to be mild, with probable relation to the trial product, and eventually reported as recovered. Mean (SD) HbA_1c_ at week 34 was 5.83% (0.45) in the somapacitan group and 5.75% (0.39) in the daily GH group.

Injection-site reactions were reported in one participant in the somapacitan group only. The injection-site reaction was considered mild and described as subcutaneous hemorrhage. No anti-somapacitan antibodies and no anti-human GH antibodies were detected in any of the participants.

## 4 Discussion

This subgroup study of the phase 3 REAL 1 trial (13), investigating the Japanese treatment-naïve participants with AGHD randomized to once-weekly somapacitan or daily GH, showed similar beneficial changes in body composition measures, tolerability, and safety in the two groups. The reductions in visceral fat are especially important, because visceral fat is well-established as an independent marker of cardiovascular and metabolic morbidity and mortality risk (15).

In the global REAL 1 trial, an apparent less pronounced effect of somapacitan compared with daily GH was seen for change in truncal fat percentage (13). This difference was not observed in the Japanese subpopulation, as the change from baseline in truncal fat percentage was close to being identical. This may be a result of the Japanese subpopulation being more homogeneous than the global trial population, and the groups having similar baseline characteristics and being comparable in terms of important determinators of GH response, such as age, sex, and oral estrogen. In the Japanese subpopulation, the point estimates for other body composition measures showed slightly more pronounced decreases in visceral fat and increases in total body weight and lean body mass measures for somapacitan compared to daily GH. However, when adjusting for baseline values, the body composition measures became more similar between the groups. The safety findings were similar to those of the global trial and were similar between the groups. Overall, the results are in alignment with the global REAL 1 trial.

An analogue example of a Japanese subgroup study (16) was recently performed in a pediatric GHD population for the pivotal REAL 4 trial (12), randomizing 200 treatment-naïve children with GHD to once-weekly somapacitan or daily GH. The Japanese subgroup included 30 participants. The study demonstrated similar efficacy, safety, and tolerability between the groups, and was in alignment with the results for the global REAL 4 trial population.

The findings of the present study are in line with another phase 3 trial, including previously GH-treated Japanese individuals with AGHD. In this study, similar, sustained effects of once-weekly somapacitan (n=46) and daily GH (n=16) after 52 weeks of treatment on abdominal adipose tissue (visceral, subcutaneous, and total abdominal adipose tissue) measured with CT scans, as well as sustained IGF-I profiles, were reported (9).

While once-weekly somapacitan and daily GH demonstrate similar efficacy and safety, once-weekly somapacitan provides important advantages over daily GH. Daily GH injections can be burdensome (6), and even more so as patients with multiple hormone deficiencies may already be taking several drugs (17). Furthermore, once-daily injections can be a barrier to treatment initiation, adherence, and continuation (6, 18). Long-term adherence is an issue and it is estimated that 20–30% of patients will discontinue GH replacement therapy either permanently or for longer periods (19). Thus, the reduced treatment burden of somapacitan may facilitate earlier treatment start, higher adherence, and lead to improved patient care for adults with GHD.

This study had several strengths. Overall, the trial can be considered well conducted and the adherence to treatment was high in the Japanese population. Furthermore, the randomization in the global trial was stratified according to region (Japan/all other countries) ensuring equal distribution of Japanese participants in each of the treatment arms. Finally, the Japanese population is generally considered very homogeneous, making it suitable to investigate in subgroup study of larger multinational trials.

This study had some limitations. The global REAL 1 trial was not powered for detecting differences between somapacitan and daily GH, and the Japanese subgroup study was further limited in number of participants. Furthermore, the participants were not blinded with respect to somapacitan compared to daily GH (the global study was only blinded with respect to somapacitan compared to placebo). Nonetheless, the results presented here provide valuable insights into the effect of somapacitan in a more homogenous subpopulation, as well as the Japanese population specifically, and further supports and expands on the findings of the global REAL 1 trial.

In conclusion, this subgroup study of the multinational pivotal phase 3 AGHD trial provides important insights into the effects of somapacitan in the Japanese AGHD population. The results show similar efficacy and safety of somapacitan administered once weekly compared to daily GH in this homogenous population of Japanese AGHD patients. This study supports results from previous studies in which somapacitan administered once weekly was as efficacious and well tolerated as daily GH in people with AGHD. A plain language summary of this work is available (20).

## Data Availability

The datasets presented in this article are not readily available because they contain confidential patient information, but some data is available from the corresponding author on reasonable request. Requests to access the datasets should be directed to MR, mhr@novonordisk.com.
