# Supplementary material for: Efficacy in Japanese adults with growth hormone deficiency receiving weekly somapacitan or daily growth hormone: results from phase 3 REAL 1 trial
Source: Front Endocrinol (Lausanne). 2025 Feb 7;16:1534891. doi: 10.3389/fendo.2025.1534891 (PMC11842232; doi:10.3389/fendo.2025.1534891)
Supplement: Supplementary file 1 [file DataSheet1.pdf]

Supplementary Material

Supplementary Table S1. Adjusted mean changes from baseline to week 34 in DXA-derived body composition measures

	**Somapacitan** (n = 18)	**Daily GH** (n = 18)
**Effects on fat mass**		
Truncal fat percentage, %		
Change	-1.98	-2.19
Percentage change in visceral fat[a]		
Change	-16.3	-4.3
Total fat mass, kg		
Change	-0.42	-0.84
Truncal fat mass, kg		
Change	-0.48	-0.45
Gynoid fat mass, kg		
Change	-0.03	-0.08
Android fat mass, kg		
Change	-0.16	-0.11
**Effects on lean mass**		
Total lean body mass, kg		
Change	1.43	1.07
Truncal lean body mass, kg		
Change	1.00	0.89
Appendicular skeletal muscle mass, kg		
Change	0.35	0.16

Values are adjusted means estimated from analysis of covariance models with treatment, GHD onset type, sex as factors and baseline as a covariate.
[a] Post hoc defined endpoint. Changes are expressed as a percentage, therefore initial values are 100%.
DXA, dual-energy x-ray absorptiometry; GH, growth hormone; GHD, growth hormone deficiency.

Supplementary Table S2. IGF-I SDS, IGFBP-3 SDS, and IGF-I/IGFBP-3 molar ratio at each visit

		Somapacitan			Daily GH		
**Visit**	**Sample type**	**Mean**	**SD**	**N**	**Mean**	**SD**	**N**
**IGF-I SDS**							
0	Trough	-2.13	1.11	18	-2.13	0.84	18
1	Average	0.06	1.09	18	-0.33	1.15	18
2	Trough	-1.08	0.98	17	-0.35	1.12	18
3	Average	0.23	1.01	18	0.02	0.96	18
4	Trough	-0.94	0.91	18	-0.14	0.94	18
5	Average	0.43	0.86	18	0.14	0.66	18
7	Average	0.52	0.87	18	0.06	0.62	18
8	Trough	-0.58	0.73	18	0.08	0.67	18
9	Average	0.60	0.81	18	0.13	0.94	18
16	Average	0.36	0.72	18	0.31	1.07	18
25	Average	0.34	0.85	18	0.13	0.76	18
33	Average	0.19	0.80	18	0.10	1.08	18
**IGFBP-3 SDS**							
0	Trough	-1.14	1.42	18	-1.13	1.24	18
1	Average	0.31	1.31	18	-0.04	1.26	18
2	Trough	-0.35	1.28	17	-0.26	1.06	18
3	Average	0.36	1.27	18	0.32	1.43	18
4	Trough	-0.32	1.23	18	-0.06	1.00	18
5	Average	0.32	0.89	18	0.21	1.06	18
7	Average	0.41	1.09	18	0.04	1.01	18
8	Trough	0.18	1.37	18	0.14	1.09	18
9	Average	0.38	1.03	18	0.16	1.19	18
16	Average	0.40	1.04	18	0.51	1.09	18
25	Average	-0.06	1.13	18	0.07	1.06	18
33	Average	0.27	1.25	18	0.14	1.25	18
**IGF-I/IGFBP-3 molar ratio**							
0	Trough	7.10	1.90	18	7.06	1.94	18
1	Average	11.16	3.12	18	10.52	3.08	18
2	Trough	8.73	1.60	17	10.88	3.06	18
3	Average	11.68	3.44	18	11.01	3.64	18
4	Trough	9.13	1.93	18	11.27	3.24	18
5	Average	12.24	2.75	18	11.64	2.93	18
7	Average	12.46	3.48	18	11.67	2.40	18
8	Trough	9.36	1.94	18	11.53	2.96	18
9	Average	12.95	3.74	18	12.01	4.66	18
16	Average	11.75	2.28	18	11.52	4.37	18
25	Average	13.12	3.11	18	11.96	3.65	18
33	Average	11.59	2.38	18	11.56	3.49	18

GH, growth hormone; GHD, growth hormone deficiency; IGF-I, insulin-like growth factor I; IGFBP-3, IGF-binding protein 3; SD, standard deviation; SDS, standard deviation score.
